# Postpartum Depression Screening in Latvia: Validation and Optimal Cut-Off of the Edinburgh Postnatal Depression Scale

**DOI:** 10.3390/medicina62040668

**Published:** 2026-04-01

**Authors:** Marija Lazareva, Lubova Renemane, Vineta Viktorija Vinogradova, Silvija Cipare, Linda Rubene-Kesele, Liva Kise, Nancy Byatt, Elmars Rancans

**Affiliations:** 1 Department of Psychiatry and Narcology, Riga Stradins University, LV-1007 Riga, Latvia; 2 Department of Obstetrics and Gynaecology, Riga Stradins University, LV-1007 Riga, Latvia; 3 Departments of Psychiatry and Behavioral Sciences, Obstetrics and Gynaecology, and Population and Quantitative Health Sciences, UMass Chan Medical School, Worcester, MA 01655, USA

**Keywords:** postpartum depression, EPDS, validation study, screening, diagnostic accuracy, Latvia, cut-off score, perinatal mental health

## Abstract

*Background and Objectives*: Postpartum depression (PPD) is a prevalent mental health condition with substantial consequences for mothers, infants, and families. The Edinburgh Postnatal Depression Scale (EPDS) is the most widely used screening instrument for PPD; however, optimal cut-off scores vary across populations, necessitating local validation. No prior study has evaluated the diagnostic performance of the EPDS against a structured clinical interview in Latvia. To assess the reliability and diagnostic accuracy of the Latvian version of the EPDS and to determine the optimal cut-off score for detecting PPD in a Latvian outpatient population 4–6 weeks after childbirth. *Materials and Methods*: A cross-sectional study was conducted at the outpatient department of Riga Maternity Hospital between June 2024 and May 2025. Women aged ≥18 years attending routine postnatal check-ups were screened using the Patient Health Questionnaire-9 (PHQ-9). Those scoring ≥5 were invited to complete the EPDS and participate in a structured diagnostic interview using the Mini International Neuropsychiatric Interview (MINI) 7.0.2. Internal consistency was assessed using Cronbach’s alpha. Receiver operating characteristic (ROC) analysis was performed to evaluate diagnostic accuracy and identify the optimal cut-off score based on sensitivity, specificity, likelihood ratios, and the Youden Index. *Results*: A total of 272 women were screened, and 101 completed the EPDS; 78.63% of screen-positive participants underwent the MINI. The EPDS demonstrated excellent internal consistency (Cronbach’s α = 0.871). ROC analysis indicated strong discriminative ability (AUC = 0.852, 95% CI 0.759–0.945, *p* < 0.001). A cut-off score of ≥11 provided the optimal balance between sensitivity (0.74) and specificity (0.82), with the highest Youden Index (0.56) and a positive likelihood ratio of 4.14. *Conclusions*: The Latvian version of the EPDS is a reliable and diagnostically accurate screening instrument for PPD 4–6 weeks after delivery. A cut-off score of ≥11 appears optimal for routine screening in Latvian outpatient settings. These findings support the integration of EPDS-based screening into structured postpartum care and underscore the value of validating screening instruments within specific cultural and clinical contexts.

## 1. Introduction

Postpartum depression (PPD) is a major depressive episode that typically develops within the first 4–6 weeks after delivery [1,2]. It is essential to differentiate PPD from the “baby blues,” a transient and self-limiting condition characterized by mild emotional instability that generally resolves spontaneously within a few weeks [3]. In contrast, PPD is defined by persistent symptoms that markedly impair a woman’s daily functioning, affect family dynamics and social interactions, and may also have detrimental consequences for the infant’s health and development [4,5].

PPD can disrupt the formation of early mother-infant bonding, which may, in turn, be associated with delays in cognitive and language development, as well as an increased risk of behavioural, psychopathological, and somatic problems in children [6,7,8,9]. Equally concerning are epidemiological findings suggesting that up to 20% of maternal deaths in the postpartum period are attributable to suicide [10]. Moreover, the profound disturbances of the mother-infant relationship in the context of depressive symptomatology may, in rare but documented cases, contribute to incidents of infanticide [6].

Rates of PPD vary substantially across studies, reflecting differences in regional, socio-economic, and methodological contexts. According to global estimates from 2021, approximately 17.22% of women worldwide experience symptoms of PPD, with lower prevalence reported in high-income countries [15.5%] and higher rates observed in resource-limited regions (19.9%) [11]. A similar gradient is evident within Europe, where prevalence is estimated at 12.91% in Western and 16.62% in Eastern countries [11]. In addition, early postpartum affective disturbances such as “baby blues”, reported in up to 39% of women in pooled estimates, have been shown to substantially increase the risk of elevated depressive symptoms across the first postpartum year—about 20% of women presenting symptoms of “baby blues” receive a diagnosis of PPD in the first 12 month after delivery [12].

In Latvia, 14,490 live births were registered in 2023 and 12,887 in 2024, reflecting a sustained decline in fertility rates over recent years [13]. Although maternal healthcare services are universally accessible and include routine antenatal and postnatal care, structured screening for perinatal mental health disorders is not yet systematically integrated into clinical practice [14]. National epidemiological data on PPD remain scarce, limiting the ability to accurately estimate disease burden and plan targeted interventions. These gaps underscore the importance of validating reliable screening instruments tailored to the Latvian population.

In addition to its significant repercussions for maternal and child health, PPD imposes a considerable economic burden, driving increases in healthcare expenditures and productivity losses that, in several settings, reach billions. For instance, in Germany, women with notable mental health or psychosocial difficulties incurred adjusted postnatal costs €1713 higher than those without such challenges [15]. In the United Kingdom, the lifetime costs associated with perinatal depression contribute to a national economic burden of £6.6 billion [16]. Collectively, these findings underscore the importance of developing and implementing effective strategies aimed at the early identification of PPD to reduce individual and family suffering and mitigate preventable healthcare-related costs.

Researchers, professional societies, and policy makers recommend that care during pregnancy and the postpartum period should encompass not only medical interventions aimed at safeguarding physical health but also systematic attention to the emotional and psychological well-being of mothers [1,17,18,19]. Consistent with this perspective, international clinical guidelines—including those issued by the National Institute for Health and Care Excellence (NICE)—recommend routine assessment of maternal mental health throughout the perinatal period [2]. Screening programmes that use brief self-report instruments improve the detection of perinatal depressive symptoms [20]. Although such tools are not designed to provide definitive diagnoses, they serve as valuable indicators of potential mental health difficulties [21], thereby supporting decisions regarding further evaluation and timely referral to specialists.

International clinical guidelines consistently underscore the importance of systematic screening for depressive symptoms throughout the perinatal period, advocating for the integration of brief, validated self-report instruments into routine obstetric and postnatal care [2,22,23]. Among the available screening tools, the Edinburgh Postnatal Depression Scale (EPDS) is the most widely implemented and extensively studied instrument specifically developed for the postpartum context [24,25]. However, despite its broad international adoption, substantial variation exists in the optimal EPDS cut-off scores across different populations and healthcare settings [26,27]. These differences are largely attributable to cultural, linguistic, and socio-economic factors, underscoring the necessity of population-specific validation.

The application of cut-off scores derived from other countries, even geographically or culturally proximate ones, has been shown to be methodologically unsound and may lead to misestimation of the true burden of PPD, with direct implications for healthcare planning and resource allocation [28,29]. Accurate calibration of the EPDS is essential to maintain an appropriate balance between sensitivity and specificity. Overly low thresholds result in excessive false-positive findings and inefficient use of healthcare resources, whereas false-negative cases carry potentially severe consequences [30,31,32]. Despite these considerations, no studies to date have systematically examined the psychometric properties and diagnostic accuracy of the EPDS against a structured clinical interview in postpartum populations in Latvia. Accordingly, our study aimed to (1) evaluate the reliability and diagnostic performance of the Latvian version of the EPDS against a structured clinical interview in an outpatient postpartum sample, and (2) establish an empirically grounded cut-off score suitable for early identification of clinically relevant PPD symptoms within Latvia.

## 2. Materials and Methods

### 2.1. Study Design and Participants

The study was conducted as part of the research project “Advancing Postpartum Depression Care in Latvia,” which sought to evaluate and improve approaches to PPD care. The objectives included validating the EPDS [24], developing an instrument to assess risk factors for PPD, and exploring strategies to enhance support for mothers affected by PPD in line with international best practices. The study employed a cross-sectional design. All women aged over 18 years who attended a routine postnatal gynecological check-up 4–6 weeks after delivery from June 2024 to May 2025 at the outpatient department of Riga Maternity Hospital—the largest maternity outpatient facility in Latvia—were eligible for inclusion if their initial Patient Health Questionnaire-9 (PHQ-9) score was 5 or higher. Women who screened positive on the PHQ-9 were invited to complete the EPDS and participate in a structured diagnostic interview; however, not all eligible participants agreed to continue participation or were reachable for follow-up assessment.

### 2.2. Data Collection

Sociodemographic variables were collected at two stages of the study. Basic characteristics (e.g., age and place of residence) were obtained during the initial screening phase from the full cohort. Subsequent data collection, including completion of the extended sociodemographic questionnaire with additional variables (e.g., education, marital status, employment, and income) and the EPDS, were collected only from participants who proceeded to the second-stage assessment and was carried out remotely. Remote data collection methods were employed to ensure maximum flexibility for new mothers and to minimize disruptions to their caregiving responsibilities. Participants could choose either to complete the electronic questionnaires independently or to do so during a structured remote video interview with a trained psychiatrist from the research team; in the latter case, participants were provided several minutes to complete the questionnaire independently, as EPDS completion typically requires approximately 3–5 min. The Mini International Neuropsychiatric Interview (MINI), version 7.0.2 [33] was administered during the structured remote video interview by a trained psychiatrist within two weeks of the initial PHQ-9 screening.

Informed consent was obtained from all participants prior to their inclusion in the study. To ensure confidentiality, each participant was assigned a unique identification code that was used throughout all stages of data processing. Data management was carried out using the REDCap platform, which provides secure storage and administration of electronic surveys [34]. Licencing and long-term data preservation were ensured by Riga Stradins University. The study was conducted in accordance with the principles of the Declaration of Helsinki and was approved by the Ethics Committee of Riga Stradins University, Riga, Latvia (No. 2-PEK-4/398/2024, dated 9 May 2024).

### 2.3. Measures

The PHQ-9 questionnaire, using the previously adapted Latvian-language version validated for use in Latvia, was employed to screen and select participants [35]. The PHQ-9 is widely recognized as a practical and reliable tool for screening for depression and depression symptom severity in both clinical settings and research. Based on the nine diagnostic criteria for a major depressive episode outlined in the Diagnostic and Statistical Manual of Mental Disorders, Fifth Edition (DSM-5) [36], the PHQ-9 asks respondents to indicate how frequently they have experienced specific symptoms over the preceding two weeks, thus providing a current measure of their emotional state. In the postpartum context, the PHQ-9 is particularly informative, because it assesses symptoms commonly associated with PPD, such as sleep disturbances, low mood, and changes in appetite. Its brevity and ease of administration make it well suited for the early identification of women who may require further psychological or psychiatric evaluation, even though it was not originally developed specifically for postpartum populations [37]. In the present study, the PHQ-9 was used as an initial screening instrument to identify participants eligible for second-stage assessment and did not serve as the diagnostic reference standard for evaluation of EPDS performance.

In addition, an extended sociodemographic questionnaire was developed for research purposes. This questionnaire collected comprehensive information on participant characteristics, including age, place of residence, educational attainment, marital status, employment situation, and income.

In clinical practice, the EPDS plays a central role as a screening instrument that facilitates the timely detection of emotional difficulties that women may experience after childbirth. The scale enables rapid screening of a mother’s psychological state by identifying symptoms such as anxiety, low mood, or feelings of hopelessness—features that often remain unrecognized without systematic screening [24]. The scale is a short 10-item self-report questionnaire that assesses core aspects of emotional well-being during the postpartum period over the preceding seven days [24]. Each item is scored on a four-point scale, yielding a total score ranging from 0 to 30, with higher scores indicating greater severity of depressive symptoms. Its concise structure makes the EPDS both user-friendly and sufficiently informative to function as a reliable tool for the early detection of postpartum mental health disturbances. Owing to its focus on the postpartum period, the EPDS demonstrates higher specificity for PPD compared to more general tools such as the PHQ-9 [38]. Our study employed a previously linguistically adapted Latvian version of the EPDS that had been developed in earlier research [39]. Prior to the main phase of data collection, a pilot assessment was conducted involving 20 women receiving postpartum outpatient care to evaluate the clarity, comprehensibility, and overall feasibility of the Latvian version of the EPDS within the clinical context.

The MINI 7.0.2 is a structured diagnostic interview designed to assess mental disorders according to DSM-5 criteria. The instrument is organized into separate modules, including those assessing depressive disorders. Each module begins with a series of screening questions; if these initial questions rule out pathology, the module is terminated, whereas positive responses prompt more detailed diagnostic questioning [33]. These subscales allow for the evaluation of depressive symptoms which may emerge during the postpartum period. The MINI employs a concise yet comprehensive structure that enables efficient identification of psychiatric disorders in both clinical and research settings while requiring minimal administration time [33]. Previous studies have shown that the MINI detects a broader spectrum of depressive cases than many other diagnostic instruments, capturing a wider range of symptomatology [40]. This capacity makes it a particularly valuable tool for diagnostic assessment, ensuring more precise and comprehensive evaluation of mental health status.

In this study, the MINI served not only as the primary diagnostic instrument for depressive disorders but also as the criterion reference for calibrating the EPDS. As the diagnostic “gold standard” [41,42], it enabled direct comparison of EPDS scores with clinically confirmed outcomes, supporting the determination of the optimal cut-off value for identifying clinically relevant PPD symptoms in the sample. Thus, the use of the MINI provided an empirical basis for refining the diagnostic threshold of the EPDS and contributed to adapting the scale to the characteristics of the local population, ultimately enhancing its diagnostic accuracy for postpartum screening.

### 2.4. Statistical Analysis

To evaluate the psychometric properties of the EPDS, we employed a comprehensive statistical framework designed to assess both the reliability and diagnostic performance of the scale. Internal consistency, a key indicator of measurement reliability, was examined using Cronbach’s alpha—a widely accepted metric reflecting the extent to which questionnaire items measure a shared underlying psychological construct.

In addition to reliability assessment, criterion validity was rigorously evaluated to determine the degree to which the EPDS accurately differentiates between the presence and absence of PPD. Receiver operating characteristic (ROC) analysis was conducted to generate sensitivity and specificity curves across a range of potential cut-off values. This approach enabled the calculation of several diagnostic indicators, including sensitivity (the proportion of true positive cases) and specificity (the proportion of true negative cases). Additional metrics were also derived, such as the positive and negative predictive values (PPV and NPV), which reflect the probability that individuals with positive or negative test results truly do or do not have the condition. Furthermore, positive and negative likelihood ratios (LR+ and LR−) were computed to quantify the diagnostic strength of each threshold, expressing how much more (or less) likely a given test result is to occur among individuals with PPD compared to those without it [43]. The Youden index (sensitivity + specificity − 1) was used to identify the value that provided the best balance between sensitivity and specificity.

The present study focused primarily on evaluating the diagnostic performance and internal consistency of the Latvian version of the EPDS in comparison with a structured clinical interview (MINI) as the reference standard. Therefore, psychometric analyses were restricted to measures of reliability and criterion validity derived from ROC analysis. Additional structural psychometric analyses, such as exploratory or confirmatory factor analysis or item-level testing, were not performed because the study was designed as a diagnostic accuracy validation rather than a full-scale construct validation study.

Data analysis was performed using International Business Machines Corporation’s Statistical Package for the Social Sciences version 29.0. Based on the ROC curves, the area under the curve (AUC) was calculated as an integrated measure of diagnostic performance, representing the probability that the scale correctly distinguishes a case of PPD from a non-depressed participant.

## 3. Results

**Figure 1:**
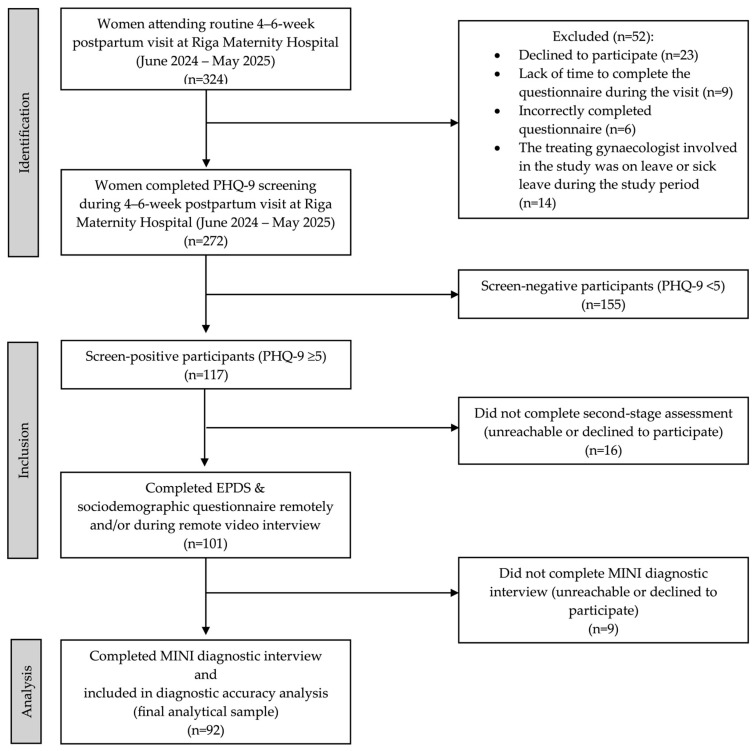
Flow diagram of participant recruitment and inclusion process.

Over a 12-month period, data were collected from 272 women aged 18 to 49 years (mean age 30.66 ± 5.59), representing 84% of all women attending their scheduled 4–6-week postpartum visit at the outpatient department of Riga Maternity Hospital. Initial screening with the PHQ-9 showed that 43.02% of participants scored ≥5, indicating the presence of depressive symptoms and a need for further evaluation. The participant recruitment and inclusion process across the different stages of the study is illustrated in Figure 1.

101 participants (86.32% among women who screened positive on the PHQ-9) completed the sociodemographic questionnaire and the EPDS. A structured psychiatric interview was completed by 78.63% of screen-positive women, accordingly 21.37% either declined participation or could not be contacted. Of those who did not complete the interview, 16% had an EPDS score ≥ 11, and the mean EPDS score within this subgroup was 14. According to the MINI diagnostic interview, PPD was confirmed in 16.24% of women who screened positive on the PHQ-9. Table 1 provides a summary of the sociodemographic characteristics of the study sample.

The Latvian version of the EPDS demonstrated excellent internal consistency, with a Cronbach’s alpha of 0.871 across the 10 items. This coefficient indicates a high degree of inter-item coherence and suggests that the scale reliably measures a single latent construct—PPD [42,43,44].

**Figure 2:**
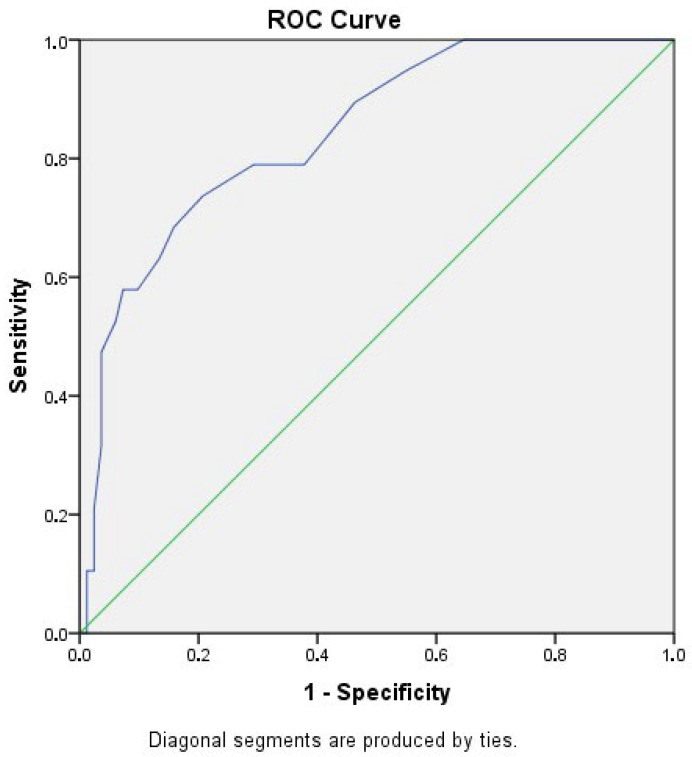
Receiver operating characteristics curve of the Edinburgh Postnatal Depression Scale.

ROC analysis demonstrated that the EPDS differentiated participants with MINI-confirmed PPD from those without the diagnosis. The ROC curve was positioned above the line of no discrimination. The area under the curve was 0.852 (SE = 0.047; 95% CI: 0.759–0.945), indicating good diagnostic accuracy. The confidence interval did not include 0.5, and the result was statistically significant (*p* < 0.001). ROC curves for EPDS are shown in Figure 2.

The analysis of the diagnostic indicators of the EPDS demonstrated that increasing the cut-off score was associated with a systematic rise in specificity and LR+, while sensitivity and LR− decreased accordingly. Lower thresholds (≥1–6) yielded maximal sensitivity but lacked sufficient diagnostic strength to confirm PPD. The most balanced diagnostic performance was observed at the cut-off ≥ 11, which provided an optimal combination of sensitivity (0.74) and specificity (0.82), the highest Youden Index (0.56), and a meaningful increase in likelihood for a positive test result (LR+ = 4.14)—all values aligned with contemporary meta-analytic evidence [27]. Higher cut-off scores were characterized by excellent specificity and substantial increases in LR+, yet these advantages were offset by a pronounced reduction in sensitivity, limiting their applicability to confirmatory diagnostic contexts rather than screening. Taken together, the cut-off ≥ 11 appears to represent the optimal threshold for identifying clinically significant depressive symptoms in this sample. The results of the ROC analysis for the EPDS in detecting PPD established by the MINI are presented in Table 2.

## 4. Discussion

Our study provides robust evidence supporting the reliability and diagnostic validity of the EPDS in a Latvian postpartum outpatient population assessed 4–6 weeks after delivery. The Latvian language version demonstrated excellent internal consistency and strong discriminative ability when evaluated against the MINI, reinforcing the suitability of the EPDS as a screening instrument for PPD in bilingual clinical settings.

The internal consistency observed in this study (Cronbach’s α = 0.871) is consistent with, and in some cases exceeds, values reported in international validation studies, where alpha coefficients typically range from 0.78 to 0.88 [21,44,45,46]. This finding indicates that EPDS items coherently assess a single latent construct related to postpartum depressive symptomatology, supporting both the stability and reproducibility of the scale and reinforcing it suitability as a reliable screening tool for PPD symptoms.

In terms of diagnostic performance, ROC analysis further demonstrated that the EPDS effectively distinguished participants with PPD established by the MINI from those without the diagnosis and the ROC curve was clearly positioned above the line of random classification. The area under the curve (AUC = 0.852) reflected a high level of diagnostic accuracy, while the small standard error (SE = 0.047) and the narrow 95% confidence interval (0.759–0.945), which did not include 0.5, confirmed the precision of the estimate. The result was statistically significant (*p* < 0.001), indicating that the discriminative ability of the EPDS is highly unlikely to be attributable to chance. Meta-analytic evidence suggests that AUC values exceeding 0.80 indicate good diagnostic accuracy for screening instruments used in perinatal populations [27,47]. Thus, our present findings align closely with international benchmarks and confirm the utility of the EPDS for identifying women at increased risk of PPD during routine postpartum care in Latvia.

A key contribution of this study is the empirical determination of an optimal EPDS cut-off score for the Latvian postpartum population. Based on ROC analysis and the Youden Index, a threshold of ≥11 provided the most balanced trade-off between sensitivity (0.74) and specificity (0.82), accompanied by clinically meaningful likelihood ratios. From a clinical perspective, the likelihood ratio associated with the optimal cut-off score (L+ = 4.14) suggests a moderate increase in the probability of PPD following a positive screening result, supporting the usefulness of this threshold for identifying women who may benefit from further psychiatric assessment. At the same time, the corresponding negative likelihood ratio (LR− = 0.32) indicates a meaningful reduction in the probability of PPD when screening results are below this threshold. Together, these findings support the role of the EPDS as an effective first-stage screening instrument within routine postpartum care, where the goal is not diagnostic confirmation but the identification of women requiring additional clinical evaluation.

This result is particularly important in light of the substantial international variability in recommended EPDS cut-off scores. Across published studies, optimal EPDS thresholds have ranged widely from as low as 7 to as high as 15, depending on population characteristics, cultural context, timing of assessment, and diagnostic reference standards. European findings demonstrate considerable heterogeneity in recommended EPDS cut-off scores, with reported thresholds ranging from 8/9 in Croatia [48] to ≥11 in Denmark [49] and 11/12 in Malta [50]. Lower cut-off scores have been reported in urban Ethiopian samples (6/7) [51], as well as in studies from Pakistan and Brazil, where thresholds of ≥8 were identified as optimal [52,53]. In contrast, considerably higher cut-off values have been recommended in Japan (≥13) [54]. These findings underscore the extent to which cultural, linguistic, and healthcare system factors influence the diagnostic performance of the EPDS.

Large-scale individual participant data meta-analyses further demonstrate that no single EPDS cut-off performs optimally across all settings [27,28]. For instance, Levis et al. [27] reported that a cut-off score of ≥11 maximized the combined sensitivity and specificity, whereas a threshold of ≥13 was more specific but less sensitive. Higher cut-off values may therefore be preferable when the aim is to identify women with more pronounced symptom levels, while lower thresholds can be used to minimize false negatives. Similar variability in optimal EPDS thresholds has been reported across different national and clinical contexts, reflecting differences in study populations, screening settings, and reference diagnostic procedures, and further supporting the need for local validation of screening instruments [55]. The identification of ≥11 as the optimal cut-off in the present study is therefore consistent with international evidence and reinforces the necessity of population-specific calibration.

International clinical guidelines reflect this variability and caution against rigid reliance on any single EPDS threshold. The NICE, for example, recommends the EPDS as a screening tool while emphasizing the importance of clinical judgement and follow-up assessment rather than sole dependence on cut-off scores [2]. Similarly, Canadian and World Health Organization (WHO)-aligned recommendations stress that screening instruments should be embedded within stepped-care models that include diagnostic confirmation and access to appropriate treatment pathways [17,23]. Within this clinical and policy framework, the cut-off score of ≥11 identified in the present study appears well suited for routine postpartum screening in Latvian outpatient settings. This threshold minimizes the risk of missing clinically significant cases of PPD while avoiding excessive false-positive results that could overburden limited mental health resources. Importantly, higher cut-off scores observed in the current data achieved excellent specificity but at the cost of markedly reduced sensitivity, suggesting that such thresholds may be more appropriate for confirmatory rather than screening purposes.

From a clinical implementation perspective, the identification of a locally validated EPDS cut-off score of ≥11 provides a practical threshold that can support the integration of structured screening into routine postpartum care pathways in Latvia, particularly during scheduled 4–6-week follow-up visits that already represent a standard point of contact between mothers and healthcare providers. In settings where systematic screening for perinatal mental health conditions has not yet been fully implemented, the use of a population-specific cut-off value may improve the consistency of case identification across healthcare providers, facilitate earlier recognition of women at increased risk of PPD, and support timely referral to appropriate mental health services [15]. At the national level, these findings may contribute to the development of standardized screening recommendations and promote closer alignment of Latvian maternal healthcare practice with international perinatal mental health guidelines. In addition, the adoption of a standardized screening threshold could contribute to strengthening collaboration between obstetric and mental health services and support the development of stepped-care approaches tailored to the Latvian healthcare context.

Overall, these findings support the routine use of the EPDS with a cut-off score of ≥11 for PPD screening in Latvia. More broadly, they contribute to the growing international consensus that EPDS cut-off scores must be locally validated and interpreted within specific clinical and cultural contexts [49,56,57]. Future research should aim to replicate these findings in larger and more diverse samples, examine longitudinal outcomes associated with different screening thresholds, and evaluate the integration of EPDS-based screening into comprehensive stepped-care models for perinatal mental health.

## 5. Limitations

Several limitations of the present study should be considered when interpreting the findings. First, the study sample was derived from a single large urban maternity outpatient facility, which may limit the generalizability of the results to postpartum populations in rural areas or other healthcare settings. Sociodemographic characteristics of the sample, including a relatively high proportion of women with higher education, stable employment, and being married, indicate that the cohort largely represented a socially and economically stable, high-functioning population. As such, the sample may not fully reflect the broader postpartum population in Latvia, particularly women with lower educational attainment, unstable employment, single marital status, or greater socioeconomic vulnerability. These factors may influence both symptom expression and help-seeking behaviour and therefore could have affected observed screening performance and prevalence estimates.

Second, not all women attending the maternity centre during the study period completed the initial screening procedure. Approximately 16% of eligible women did not complete the baseline PHQ-9, and among those who screened positive, around one-fifth did not proceed to complete the EPDS and the sociodemographic questionnaire. Although overall participation rates were high, the inability to assess the entire target population may have introduced selection bias. Women who were not screened, or who declined further participation, may have differed systematically in symptom severity, help-seeking behaviour, or other relevant characteristics. This has implications not only for the interpretation of screening performance but also for the implementation of routine screening programmes, highlighting the need to better understand and address potential patient- and provider-level barriers to participation.

Third, inclusion in the extended assessment phase was restricted to women who screened positive on the PHQ-9 (score ≥ 5) at baseline. While this approach was appropriate for the objectives of the broader research project and enhanced feasibility, it may have led to an underrepresentation of women with very mild or subthreshold depressive symptoms. Consequently, prevalence estimates, and predictive values derived from this sample should be interpreted with caution and may not fully reflect the distribution of depressive symptoms in the general postpartum population.

An additional limitation relates to the possibility of partial verification bias resulting from the two-stage study design. Only participants who screened positive on the PHQ-9 (score ≥ 5) proceeded to structured diagnostic assessment using the MINI. As a result, diagnostic verification was not performed among women with lower PHQ-9 scores, which may have influenced estimates of diagnostic performance derived from ROC analysis. This approach may have led to some overestimation of sensitivity, specificity, and the area under the curve (AUC). Therefore, the reported diagnostic indicators should be interpreted with consideration of the stepwise screening design and the characteristics of the analytical sample.

Fourth, although the MINI was used as a structured diagnostic reference standard, diagnostic interviews were completed by approximately two-thirds of screening-positive participants. Non-participation in the interview phase may have introduced selection bias, particularly if women experiencing more severe symptoms were either more or less likely to engage in diagnostic assessment.

In addition, EPDS data were collected remotely, either through independent completion by participants or during a structured remote video interview with a trained psychiatrist. Although this approach increased feasibility and accessibility for postpartum women and reduced the burden associated with in-person participation, variability in response conditions may have affected the consistency of questionnaire completion. Furthermore, while structured diagnostic interviews were conducted by trained psychiatrists using the MINI, remote administration in a video interview format allowed partial observation of non-verbal behaviour but still provided fewer opportunities for comprehensive in-person clinical assessment and may therefore have influenced diagnostic precision to some extent. As a self-report screening instrument, the EPDS is not intended to replace clinical psychiatric evaluation but rather to support the early identification of women at increased risk of PPD; these factors should be considered when interpreting the findings.

Finally, the cross-sectional design of the study precludes conclusions regarding the temporal stability of EPDS scores or their predictive validity for longer-term maternal mental health outcomes. Postpartum depressive symptoms may fluctuate over time, and optimal cut-off values could differ at later postpartum stages.

Future studies incorporating qualitative methods or culturally sensitive measures could provide additional insight into these aspects.

## 6. Conclusions

Our study demonstrates that the EPDS is a reliable and diagnostically accurate screening instrument for PPD in a Latvian outpatient population assessed 4–6 weeks after childbirth. The Latvian version showed excellent internal consistency and strong discriminative validity when compared with a structured diagnostic interview, with a cut-off score of ≥11 providing the optimal balance between sensitivity and specificity. These findings align with international evidence highlighting population-specific variability in EPDS thresholds and underscore the importance of local validation. The results support the routine use of the EPDS within structured postpartum care pathways in Latvia to facilitate timely identification of women at risk for clinically significant depressive symptoms and appropriate referral for further assessment and care.

## Figures and Tables

**Table 1:** Sociodemographic characteristics of the study population among women attending the outpatient department of Riga Maternity Hospital 4–6 weeks postpartum.

Variables		n	%
Age	18–25	52	19.1%
	26–30	80	29.4%
	31+	140	51.5%
Place of residence	Riga	206	75.7%
	Rural area	32	11.8%
	Another city	34	12.5%
Mother’s education ^1^	Higher and incomplete higher	80	79.2%
	Secondary/incomplete secondary	10	9.9%
	Vocational (e.g., nursing assistant, kitchen assistant)	11	10.9%
Marital status ^1^	Never been married/lived with partner	2	2.0%
	Married, but living separately	1	1.0%
	Married/living with partner	98	97.0%
Mother’s Employment	Employed/Self-employed/Other	221	81.3%
	Unemployed	48	17.6%
	Economically inactive	3	1.1%
Partner’s employment	Employed/Self-employed/Other	227	94.6%
	Unemployed	12	5.0%
	Economically inactive	1	0.4%
Income ^1^	≥1100 and difficult to say	55	54.4%
	500–1100	43	42.6%
	No income and <500	3	3.0%

Variables collected during the initial screening stage are presented for the full screened cohort (n = 272; n = 240 for partner’s employment). ^1^ Variables obtained during the second-stage assessment are presented only for participants who completed the EPDS and extended sociodemographic questionnaire (n = 101).

**Table 2:** The ROC analyses of the EPDS Latvian version for the diagnosis of postpartum depression established by the MINI.

Cut-Off	Sensitivity	Specificity	PPV	NPV	LR+	LR−	Youden Index
EPDS ≥ 1	1	0.01	0.21	1.00	1.01	0	0.01
EPDS ≥ 2	1	0.03	0.21	1.00	1.03	0	0.03
EPDS ≥ 3	1	0.08	0.22	1.00	1.09	0	0.08
EPDS ≥ 4	1	0.18	0.24	1.00	1.22	0	0.18
EPDS ≥ 5	1	0.29	0.27	1.00	1.40	0	0.29
EPDS ≥ 6	1	0.37	0.29	1.00	1.59	0	0.37
EPDS ≥ 7	0.95	0.46	0.32	0.97	1.77	0.11	0.41
EPDS ≥ 8	0.89	0.55	0.34	0.95	1.98	0.19	0.44
EPDS ≥ 9	0.79	0.64	0.37	0.92	2.22	0.33	0.43
EPDS ≥ 10	0.79	0.74	0.44	0.93	3.03	0.28	0.53
EPDS ≥ 11	0.74	0.82	0.52	0.92	4.14	0.32	0.56
EPDS ≥ 12	0.68	0.85	0.54	0.91	4.54	0.37	0.53
EPDS ≥ 13	0.63	0.88	0.57	0.90	5.12	0.42	0.51
EPDS ≥ 14	0.58	0.92	0.65	0.89	7.04	0.46	0.50
EPDS ≥ 15	0.58	0.94	0.73	0.90	10.57	0.45	0.52
EPDS ≥ 16	0.53	0.96	0.77	0.89	12.81	0.49	0.49
EPDS ≥ 17	0.47	0.97	0.82	0.88	17.29	0.54	0.45
EPDS ≥ 18	0.32	0.97	0.75	0.85	11.53	0.70	0.29
EPDS ≥ 19	0.21	0.97	0.67	0.83	7.68	0.81	0.18
EPDS ≥ 20	0.16	0.97	0.60	0.82	5.76	0.87	0.13
EPDS ≥ 21	0.11	0.97	0.50	0.81	3.84	0.92	0.08
EPDS ≥ 22	0.11	0.99	0.67	0.81	7.68	0.91	0.09
EPDS ≥ 23	0.11	0.99	0.67	0.81	7.68	0.91	0.09
EPDS ≥ 24	0.05	0.99	0.50	0.80	3.84	0.96	0.04
EPDS ≥ 25	0	0.99	0	0.79	0.00	1.01	−0.01
EPDS ≥ 26	0	1		0.79		1	0
EPDS ≥ 27	0	1		0.79		1	0

PPV, positive predictive value; NPV, negative predictive value; LR+, positive likelihood ratio; LR−, negative likelihood ratio; ROC, receiver operating characteristics; MINI, Mini International Neuropsychiatric Interview; EPDS, Edinburgh Postnatal Depression Scale.

## Data Availability

The original contributions presented in this study are included in the article. Further inquiries can be directed to the corresponding author.

## Abbreviations

The following abbreviations are used in this manuscript:

PPD	Postpartum depression
NICE	National Institute for Health and Care Excellence
WHO	World Health Organization
EPDS	Edinburgh Postnatal Depression Scale
PHQ-9	Patient Health Questionnaire-9
MINI	Mini International Neuropsychiatric Interview
DSM-5	Diagnostic and Statistical Manual of Mental Disorders, Fifth Edition
ROC	Receiver operating characteristic
PPV	Positive predictive value
NPV	Negative predictive value
LR+	Positive likelihood ratio
LR–	Negative likelihood ratio
AUC	Area under the curve
